# Tartaric acid as a novel additive for approaching high-performance capacity retention in zinc-ion battery

**DOI:** 10.1038/s41598-022-13897-5

**Published:** 2022-08-03

**Authors:** Erfan Molaei, Mohammad Mahdi Doroodmand, Ruhollah Shaali

**Affiliations:** Department of Chemistry, Shiraz University, Shiraz, 71454 Iran

**Keywords:** Chemistry, Engineering

## Abstract

Among the rechargeable batteries, aqueous zinc-ion batteries (*ZIBs*), due to their safety, low cost, eco-friendly, and simplicity in construction, have received much attentions. One of the most critical parts of the battery technology is the electrolyte additives, which have been less studied against their essential roles. To develop the quality of these batteries, specific parameters such as economics, easy design, significant time duration, high electrical discharge, fast charge/discharge rate, acceptable power/ energy density, and acceptable cycle efficiency are essential. In this report, is focused on the aqueous solution of some white crystalline organic acids as novel electrolyte additives such as succinic, tartaric, citric, maleic, and/or acetic acids as battery over-voltage reducing agents to modify the electrical performance of the ZIBs. For instance, significant characteristics of tartaric acid as specially selected electrolyte additive to the ZIBs, exhibit an excellent capacity up to 374 mAh g^−1^ with acceptable rate capability and high-capacity retention as large as 91.0% after 7200 cycles. To investigate the battery behavior and propose the probable mechanism behind this phenomenon, some analytical methods are utilized.

## Introduction

Non-renewable energy sources such as fossil oil, natural gas, and coal are declining gradually^1^. Also, environmental pollution is becoming so severe that humans should go into new pathways to develop renewable and environmental-friendly energy sources^1^. At this condition, lots of rechargeable battery technologies are present such as traditional lead-acid batteries (based on conversion reactions) and Li-ion batteries (according to different processes, especially intercalation)^2^. The inherent limitations of these systems impede their applications at large-scale energy storage, limited energy density, poor charge/discharge efficiency, self-discharge, poor coulombic efficiency, and environmentally threatened^3–5^.

In recent years, a series of “*Aqueous Rechargeable Batteries*” (*ARBs*) were invented^6^. These ARBs are operated based on layer-by-layer electrochemical intercalation/storage of some ionic species such as Na^+^, K^+^, Mg^2+^, and Zn^2+^ from the aqueous electrolyte onto the electrode structure^6–9^. These batteries have been considered a promising energy source, due to their inherent safety**,** higher ionic conductivity, materials abundance, and environmentally benign^6–9^. Among the ARBs, Zn-ion batteries (*ARZIBs*) have proven to be the eco-friendliest energy storage systems, as they use zinc as the anode. Zinc ion batteries received extensive interest based on the multivalent characteristic that is widely available on the earth’s crust, low-cost, non-toxic aqueous electrolyte, safety, and long cycle life^10–12^. Many compounds with tunnel-type and layered-type structures enable the insertion/extraction of the Zn^2+^ ions into/from their hosts owing to the small ionic radii of the Zn^2+^ (0.74 Å)^13^. In these systems, *“Feiyu Kang's”* group showed for the first time the reversible Zn-ion insertion into the tunnel structure of the α-type MnO_2_ host, was adopted as the cathode in a ZIB^14^.

The MnO_2_ has been widely investigated as an electrode material for applications in both supercapacitors and batteries, because of its abundante availability, low cost, and environmentally friendly^15^. This compound possesses several different crystallographic forms, such as *α, β, γ, δ, λ*, and ramsdellite-type^15, 16^.

It should be considered that aqueous ZIB has major deployment challenges. These trials majorly consist of i) low chemical stability and electrochemical stability of the electrolyte, and ii) formation of zinc dendrites during cycling, corrosion, passivation, and “*Hydrogen Evolution Reaction*” (HER). These are mainly reflected in the restricted voltage windows, capacity and stability of the ZIBs. These challenges are also considered as the main factors, limited the energy density, battery recyclability, and the decomposition of electrolytes, which can cause danger of battery deformation and swelling.^17–19^.

Current research in battery technologies is focused on the exploration of the anode/cathode materials and liquid-free electrolyte materials during the optimization of the electrode/electrolyte interface^20^. Many outstanding types of research in this aspect have been done, such as the widening of the electrochemical window of electrolyte^21^, electrostatic shell protection of the electrolyte on the anode side, and cathode/electrolyte ion balance control^22^. But, in new research on ARBs, additives are being used as a low-cost solution and a practical, stable, and durable way to solve the zinc ion battery problem^17^. However, recent studies on the ZIBs have been majorly focused on cathode materials and Zn ion intercalation mechanisms^23^. For instance, to solve the issues of the Mn-based cathode, several strategies have been proposed, such as surface coating^24^, microstructure construction^25^, doping^26, 27^, electrolyte regulation^22^, etc. Fluorine doping is considered one of the newest methods for fabricating a stable and reversible framework of the Mn-based cathode, which has been widely used in electrode materials of electrochemical energy storage^28^.

However, one part of the battery, which possessed less attention during the last decades, is the cathodic/anodic over-voltage reducing species that are often considered as the electrolyte additives to the battery’s electrolyte medium to enhance the cell’s performance^29^.

To overcome the cell’s over-voltage and have significant improvement in the battery’s figures of merit, such as the reversibility, durability, and capacity, some electrolyte like tartaric, maleic, succinic, and citric acid are adopted as the electrolytes additives to access the mentioned goal. This process also is considered as one of the most cost-effective and effective methods for of the improvement the battery’s performance^29^. To solve these problems, herein, we modified the performance of the reversible aqueous Zn/MnO_2_ battery using some white crystalline organic acids as battery over-voltage reducing agents. In this system, briefly, the γ-MnO_2_ cathode was highly reversible and stable in a ZnSO_4_ aqueous electrolyte with the aqueous solutions of white crystalline organic acids such as tartaric acid as effective electrolyte additives. The motivation behind this study, therefore, creates a more efficient, reliable, environmentally friendly, low cost, high enough electrical energy, higher power densities, more improved reversibility, and longer cycle life, compared to the generally commercialized ZIBs.

## Experimental

### Materials

All the reagents were from their analytical grades. Potassium permanganate (KMnO_4_, purity: 99.0%, w/w) was purchased from Interchem U.K. Company. Manganese(II) chloride (MnCl_2_, > 99%) was related to the Sigma Aldrich Company. Activated carbon black (active surface area: 520 ± 7m^2^ g^−1^, pore diameter: 3.0 nm cm^−2^, pore volume: 02.417 ± 0.0021 m^3^ g^−1^ and purity: > 98%) was purchased from the Merck Company. In addition, polyvinylidene fluoride (*PVDF*, > 99.5%) adopted from EXIR GmbH Company. Zinc foil sheet (thickness: 0.5 mm) with purity: > 99.9 was obtained from Fulad Technology Company (Iran, Tehran). Compounds such as *N*-Methyl-2-pyrrolidone (NMP, 99.5%), zinc sulphate (ZnSO_4_, 99%), succinic acid (C_4_H_6_O_4_, > 99.5%), maleic acid (C_4_H_4_O_4_, 99%), tartaric acid (C_4_H_6_O_6_, > 99.5%), citric acid (C_6_H_8_O_7_, > 99%), acetic acid (CH_3_COOH, > 97%), glucose (C_6_H_12_O_6_, 98%), NaOH (> 99.5%) and commercial HCl (37%, W/W) were all purchased from Merck Company. Carbon fiber sheet (Carbon fiber + Epoxy Resin, resistivity: 0.50 ± 0.02 Ω cm, dimension: 5.0 × 5.0 cm, thickness: 2.0 mm, pattern: Plain) was purchased from Ltd./Pvt.Ltd. Company (India). The glass fiber sheet with 1.1 ± 0.1 mm thickness, 1.0 × 1.0 cm dimension as well as Zn foil (thickness: 0.5 mm, dimension: 1.0 × 1.0 cm) was purchased from the Saba Battery Company (Tehran, Iran).

### Analytical methods and instruments

The γ-MnO_2_ synthesis and electrochemical analytical characterizations were performed by methods such as X-ray diffraction (XRD, Bruker, Type D8- ADVANCE), electrochemical impedance spectroscopy (EIS, µ3AUT70980), cyclic voltammetry (CV, model-µ3AUT70980), field emission-scanning electron microscopy (FE-SEM, JSM-7610F Schottky Field Emission Scanning Electron Microscope, 25.0 kV, JOEL), battery tester (Hioki model: BT 3554), pH meter (Metrohm, 827 pH lab), Brunauer–Emmett–Teller (BET, Functional Capability, NOVA Company, Japan), and X-ray photoelectron spectroscopy (XPS, XPRT Kratos—AXIS Nova, Kratos Analytical Ltd., Shimadzu, Japan).

### γ-MnO_2_ synthesis

**Figure 1:**
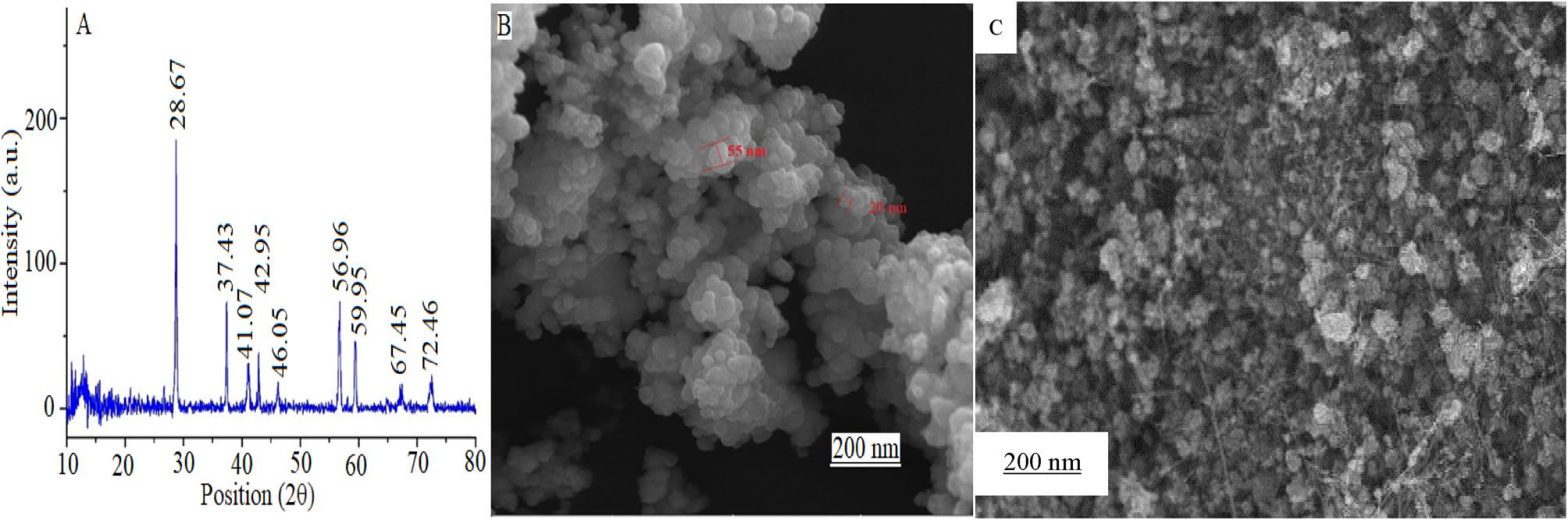
Structural and morphological characterization of γ-MnO_2_ including (**A**) X-ray diffraction pattern as well as (**B,C**) FE-SEM images of the fresh sample (γ-MnO_2_) and that modified on the cathode, respectively.

A total of 0.050 ± 0.001 mol KMnO_4_ and 0.150 ± 0.001 mol MnCl_2_ were dissolved, separately, in 100.0 mL triply distilled water (pH: 7.0 ± 0.1, specific conductivity: 0.7 μS cm^−1^, Combined Cycle Gas Center, Shiraz, Iran). Subsequently, the KMnO_4_ solution was dropped slowly into the MnCl_2_ solution with the rate of 5 drops per min during stirring (speed: 500 rpm) at room (25 oC) temperature based on the procedure reported in Ref.^30^. The product was the filtered using a filter paper (Whatman^®^ qualitative filter paper, Grade 1, Merck company), washed with triple-distilled water (100.0 mL) three times, and then dried at 100 ± 2 °C inside a thermal oven (Memmert, Incubator Oven INB200, Akribis, UK) for two days before annealing at 200 ± 2 °C for 24 h using the oven. The XRD pattern (Fig. 1A) shows the crystalline phase of γ-MnO_2_ (About the PDF standard cards, see Supporting Information). The FE-SEM image shown Fig. 1. B confirms the nano-sized structure of the synthesized γ-MnO_2_ nanoparticles with diameter between 20 and 100 nm.

Because of the safety, low cost, and better stability of each anode and cathode inside the aqueous solution, mid acidic pH was selected^31^. The pH 4.0 ± 0.1 condition has been chosen as optimum pH using HCl or NaOH standard solutions with 0.1 mol L^−1^ concentration, due to its moderate acidic conditions and the minimum anode corrosion, which were promoted especially at high acidic electrolyte solutions^14^.

### Cathode and electrolyte preparation

The γ-MnO_2_ cathode was prepared by mixing synthesized γ-MnO_2_ powder, carbon black, and polyvinylidene fluoride at the optimum weight ratio of 7.0:2.0:1.0 with total mass the same as 10.00 ± 0.1 mg cm^-2^ inside 5.0 mL of *N*-methyl pyrrolidone as solvent inside a Cole-Parmer watch glass (12/pk, Universal Scientific Industrial (Shanghai Co., Ltd. Company, China). These compounds were then mixed and homogenized using a stainless steel spatula to provide a pasty slurry mixture. This composite was then coated (immobilized) onto one side of the carbon fiber sheet handy using the spatula at room temperature. To have a regular and reproducible coating, the paste composite was scrapped vigorously long the carbon fiber sheet at least three times. After preparing the cathode support, it was put inside the oven at 140 °C for 8.0 h inside the air atmosphere to evaporate the solvent, dry the reagent and provide robust connectivity between the carbon-based cathode support and the introduced composite material. The electrolyte for this test was the mixture of 3.5 mL of triple-distilled water and 1.0 mL ZnSO_4_ solution (2.0 mol L^−1^), along with individual introduction of the aqueous solutions (0.5 mL, 1.0 mol L^−1^) of different white crystalline organic acids such as tartaric, maleic acid, citric acid, and succinic acids to the electrolyte solution.

### Procedure

A modified Zn/MnO_2_ battery system was assembled using the γ-MnO_2_-modified electrode as the cathode, a glass fiber sheet as the separator, and the Zn foil as the anode electrode. The electrolyte mixture of 3.5 mL triply distilled water and 1.0 mL ZnSO_4_ solution (2.0 mol L^−1^) and 0.5 mL aqueous solution of tartaric acid as additive with 1.0 mol L^−1^ concentration. The analyses were evaluated at the selected pH value. The OCP test was also measured by the electro analyzer. The characterization process was also based on analytical methods such as “*Brunauer–Emmett–Teller, BET*,” electrochemical impedance spectroscopy, scanning electron microscopy, cyclic voltammetry, *X-ray* photoelectron spectroscopy, and *Tafel* analysis were utilized.

## Results and discussion

Among the rechargeable battery, the aqueous ZIB, due to possessing different advantageous such as safety, cost-effective, availability, eco-friendly, and simplicity in construction, has received lots of attention. These characteristics have made the ZIB to be considered an appropriate candidate for various applications^3^. For this purpose, many scientists worldwide have focused on the ZIB features to enhance its capacitive performance. Initial attention have been focused on some general aspects of the battery technology, such as electrolyte type, and concentrations, electrode geometry and modified species on the electrode surface. Fortunately, for least two last decades, these features have significantly improved in the capacity, lifetime, electrical stability, and durability of the ZIB^6^.

However, less attention has been applied to the enfluential role of physicochemical phenomena, affecting the over-voltage of the electrode half-reactions. These attentions have been focused on different phenomena^5, 6^. For instance, electrode morphology is vital to change the electrode system pathways towards less positive/negative anodic/cathodic half-reactions, respectively. These factors also limited the polarization challenge via inter-cell ohmic potential (*IR*) reduction by controlling the electrode surface area dimension^5^. These phenomena consequently prevent the ZIBs from any high current flow of shock. However, these features are besides the promotion of the electrical conductivity of the electrode materials, controlling their morphology and lowering the inter-electrode distance^5, 6^. At these conditions, therefore, great applications of depolarizers are limited to neither the kinetic polarization nor reducing the electrolyte ohmic resistance^5^. However, to develop these systems, scientists have intensely focused on the inert ionic species, nanostructures, different surfactants (to adjust the electrolyte surface tension), etc.^5, 6^. Nevertheless, to the bests of knowledge, no reports have been published on the phenomena, during smartly and reversibly playing with the electrode active surface area. For this purpose, in this research, fantastic features of the ZIBs have been reported by focusing on different materials, as evaluated in detail in the following sections. It should be noted that, because of better chemical stability (i.e., minimum anode corrosion) of each anode and cathode electrode inside the aqueous solution, moderate acidic pH condition (4.0 ± 0.1)^14, 22^ has been selected as the optimum value.

### Approach the smartly and reversibly over-voltage reducing agents

To introduce smartly and reversibly over-voltage reducing agents, characteristics such as partial adsorption properties, moderate solubility inside the aqueous electrolyte solution, and preferential electrode’s surface intercalation properties seemed necessary. To access these reagent(s), white crystalline organic acids were considered as initial candidates.

**Figure 2:**
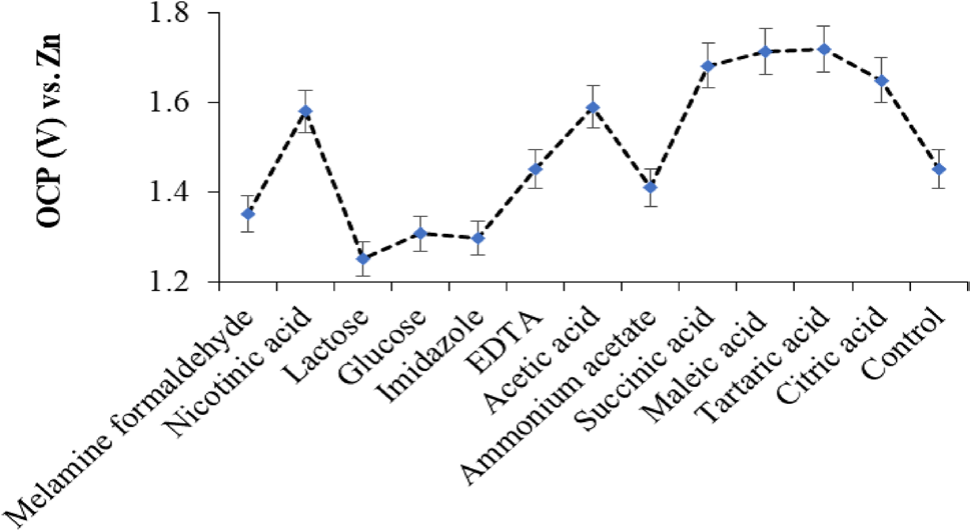
Effect of some external additives (1.0 mol L^−1^) on ZIB during its introduction to the mixture of ZnSO_4_ (2.0 mol L^−1^) and triply-distilled water at room temperature. Data are the average of four replicate analyses. Error bars: ± standard deviation.

According to our preliminary tests, different white crystalline organic acids, as electrolyte additives, were evaluated on the OCP (open circuit potential) response, according to the recommended procedure. Based on the results, significant enhancement (~ 20%) was observed in the OCP, compared to the control system (ZIB electrolyte) as well as other water-soluble organic compounds such as EDTA, glucose, etc. (Fig. 2). This phenomenon provided a significant impact on the battery's OCP.

**Figure 3:**
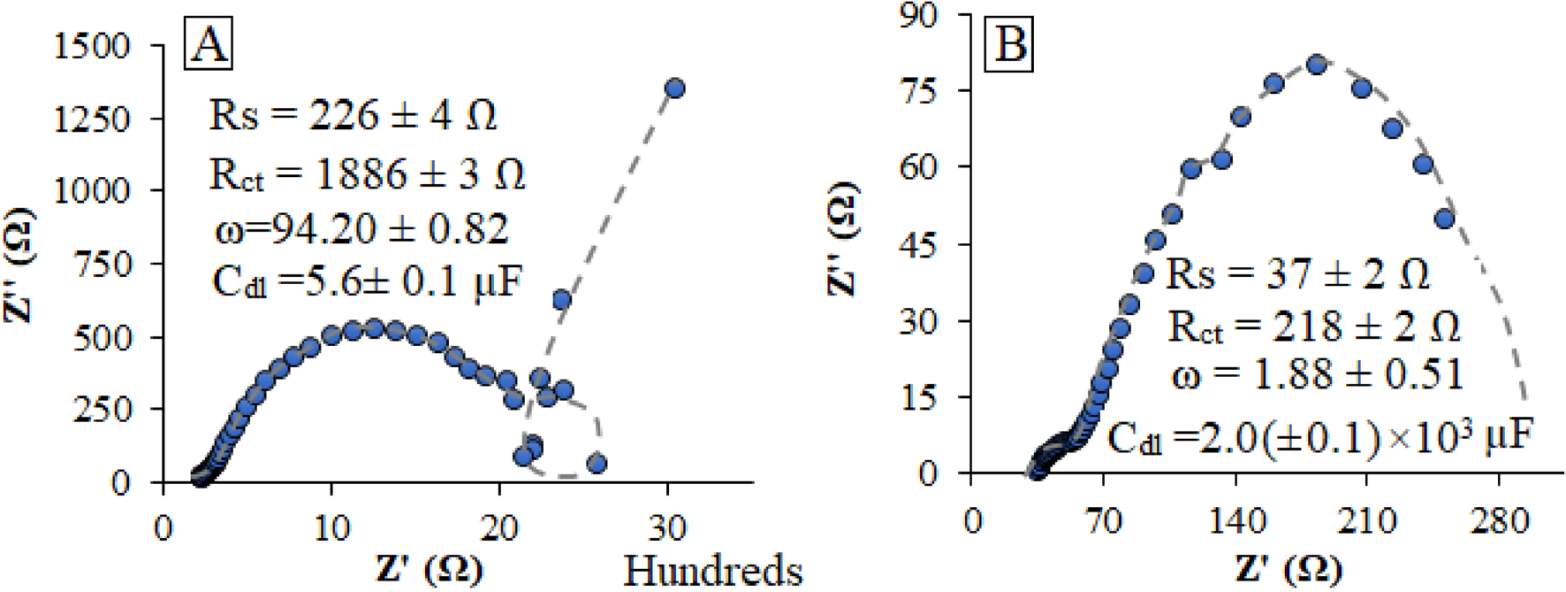
Nyquist plots of the γ-MnO_2_ cathode, zinc anode, and triply-distilled water/ZnSO_4_ 2.0 mol L^−1^ electrolyte in the (**A**) absence and (**B**) presence of tartaric acid (1.0 mol L^−1^) as an external additive at room temperature. Insets: calculated EIS parameters based on formulas reported in Ref.^32^.

As shown, maximum OCP (vs. Zn) was observed for our available 4–6-C carboxylic acids such as tartaric acid, maleic acid, and citric acids. Consequently, the aqueous solution of tartaric acid with a 1.0 molar concentration was selected for future analyses. According to the results )Fig. 3(, effects of the tartaric acid as the external electrolyte additive on some EIS (electrochemical impedance spectroscopy) parameters such as *R*_*s*_ (solution resistance), *R*_*dl*_ (double-layer resistance), and *C*_*dl*_ (double-layer capacitance) have been evaluated based on the Nyquist plots (Figs. 3A,B). Based on the formulas reported in Ref.^32^, the estimated parameter’s values were shown in the insets of Fig. 3. The coordinate unit is based on the ohm (multiple in hundred zʹ).

As clearly shown, significant reductions were observed in the *R*_*s*_ and *R*_*ct*_ of the electrolyte solution during the introduction of tartaric acid as an additive. These reductions from one side were attributed to the decrease in the *IR* drop (ohmic potential) of the cell, and from the other side, exhibited a significant increase in the K_app_ (apparent rate constant) value^33^, which was in the reverse relationship with the C_dl_^25^. Therefore, higher ion conductivity occurred. So, to evaluation of tartaric acid addition in the battery electrolyte, the ionic conductivity was determined by σ = $\frac{L}{A\times {R}_{s}}$, where L was the electrode distance and A the area of stainless-steel electrode, and R_s_ as electrolyte resistance (it should be noted that to better observation of tartaric acid effect, this experiment was performed in the 2.5 cm distance between electrodes, and 1 cm^2^ surface area of electrodes). According to the bulk resistance in the presence and absence of tartaric acid, the ionic conductivity was obtained at 62.5 and 11 mS/cm, respectively. According to the obtained results, significant increase in ionic conductivity of battery electrolytes were observed in the presence of tartaric acid. This enhancement resulted in a decrease in the resonance frequency and, consequently enhancement in the C_dl_ value. All these effects led to the significant reduction in the cathodic over-potential of the ZIBs and, consequently promotion of the electromotive force (*E*_*emf*_) of the battery from + 1.44 to + 1.75 V (vs. Zn). In addition, the higher stability of the battery at high cycles (up to at least 7200 cycles) revealed the presence of significant delay in the polarization of the cell during the introduction of the electrolyte additive. All these observations pointed to the practical roles of these white crystalline organic acids on the battery performance, which revealed the importance of the characterization of these compounds.

### Electrochemical studies

**Figure 4:**
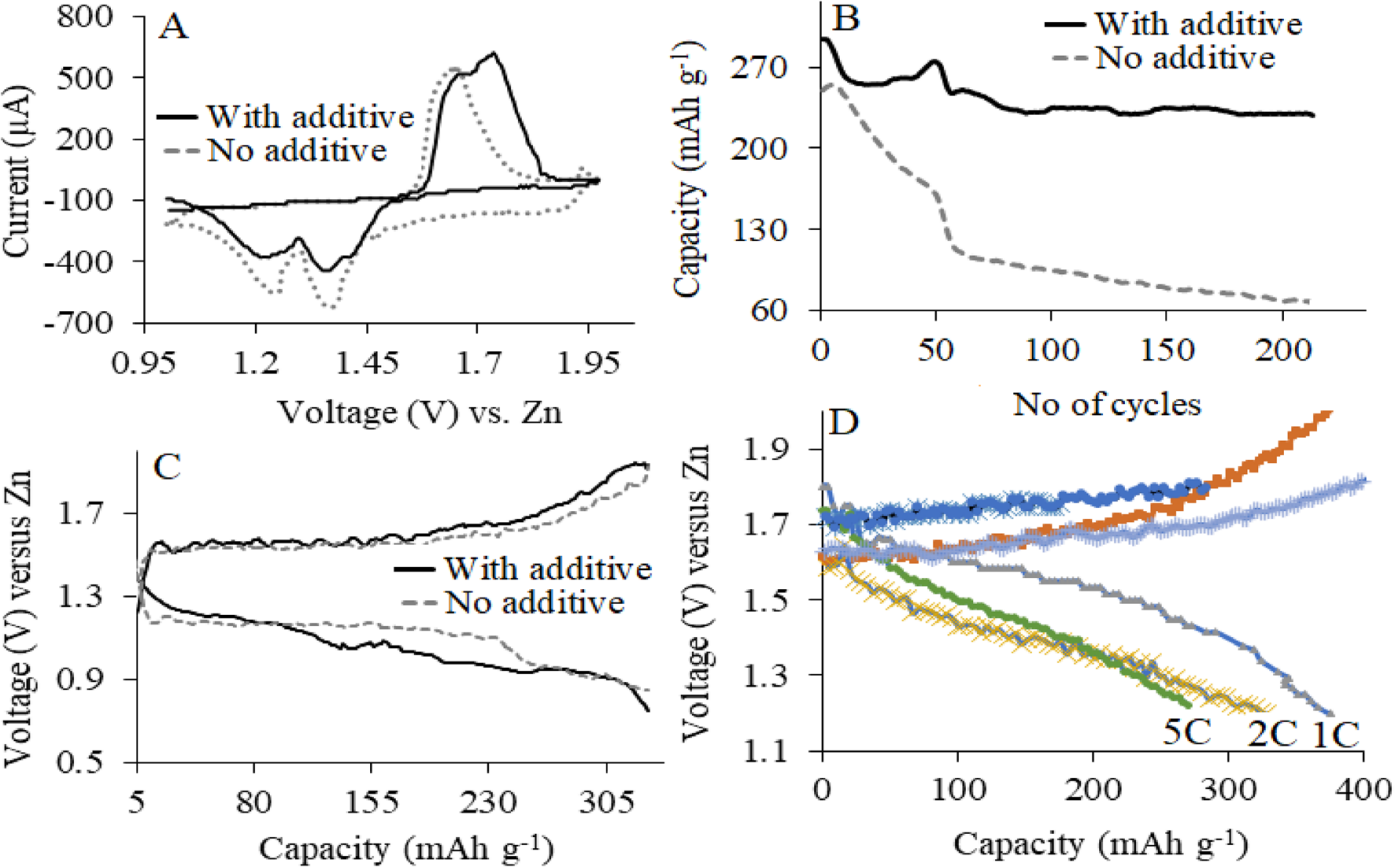
(**A**) Cyclic voltammograms, (**B**) capacity vs. no. of cycles, (**C**) voltage vs. capacity, and (**D**) capacity retentions at different current rates. Conditions: γ-MnO_2_ cathode, and zinc as an anode in the presence and absence of tartaric acid (1.0 mol L^−1^) as the electrolyte additive. Electrolyte solution: triply distilled water/ZnSO_4_ (2.0 mol L^−1^) electrolyte at room temperature.

Cyclic Voltammetry (*CV*) curves (Voltammogram, Fig. 4A), were obtained using the fabricated Zn/γ-MnO_2_ cell within the potential range between 1.0 and 2.0 V (vs. Zn) at a sweep rate of 100.0 mV s^−1^, in the presence and absence of tartaric acid as the electrolyte additive, revealed the similar redox reactions. This evidence, therefore, indicated thats tartaric acid, as the electrolyte additive, did not affect the redox reactions in the battery cell. For more confidence, the two plateau in the charge/discharge curves (Fig. 4B) approved the cycling performance of the battery and also showed significant improvements compared to the previous reported researches^14, 16^. To compare the effect of the tested electrolyte additives, the battery’s capacity was measured in the presence and absence of tartaric acid as an additive (Fig. 4C), which illustrated that; the capacity was significantly high enough compared to the lack of any additives. As exhibited, when using additive in the battery electrolyte, it was observed that the capacity vs. cycles were more improved compared to the absence of the additive that exhibited no sharp drop in the capacity curve. As mentioned, the presence of additives significantly improved the cyclability and cycle life of the battery. Based on these results, these additives not only reduced the irreversible capacity, but also protected the cathode material from the overcharge. These results were beside their influenced some physical properties of the electrolyte, such as ionic conductivity, viscosity, and wettability to the separator.

Adding tartaric acid as an additive to the electrolyte greatly improved the delivery of a higher capacity versus that tested without a tartaric acid additive. Decent capacities of 374 mAh g^-1^ were delivered for cells at the current rate of 1C, which was majorly (35%) higher than previously reported articles^2^. Figure 4D also shows the Zn/MnO_2_ battery with the tartaric acid as an additive that revealed an excellent rate capability, achieving high capacities of 327 and 270 mAh g^−1^ at 2C and 5C, respectively. The Zn/*γ*-MnO_2_ battery with tartaric acid as additive, therefore, exhibited excellent long-cycle stability, with high-capacity retention. Figure 4C indicated that the adopted additive used in this system was very promising for approaching high-performance ZIBs. This phenomenon caused to have an average operating voltage at around + 1.46 V (vs. Zn) and reversible capacity up to 340.0 mAh g^−1^ (MnO_2_) at C/5 (during a 5-h discharge) in the initial two cycles. Compared to other reports^2^, it was considered as a remarkable improvement.

The CV curves reveal two peaks at around 1.30 and 1.60 V vs. Zn^2+^/Zn. The two consistent peaks at 1.3 and 1.4 V vs. Zn^2+^/Zn showed the Zn-insertion into the γ-MnO_2_ host and the consequent reduction of Mn (IV) to the Mn (III)/Mn (II) states, which agrees with Ref.^14–20^. Similarly, the appearance of a peak and a shoulder at around + 1.60 and + 1.70 V vs. Zn^2+^/Zn, respectively, corresponded to Zn-extraction from the γ-MnO_2_ cathode as the Mn (III)/Mn (II) states undergo oxidation to the Mn (IV) state. This evidence was probably pointed to the proposed behavior (a mechanism) of the adopted additive during the promotion of the efficiency of the modified ZIB.

According to the other previously reported and published references^14–18^, partially, all types of MnO_2_ often fade their capacity sharply, and it is a traditional phenomenon in many aqueous rechargeable MnO_2_ batteries. According to the obtained results in Fig. 4B, it seems that in the absence of tartaric acid, Mn^2+^ is dissolved, and the γ-MnO_2_ structure was changed. On the other hand, the existence of tartaric acid can suppress this process and fix the Mn^2+^ in the γ-MnO_2_, and consequently, the interface of the cathode convert to quasi changeless phase.

**Figure 5:**
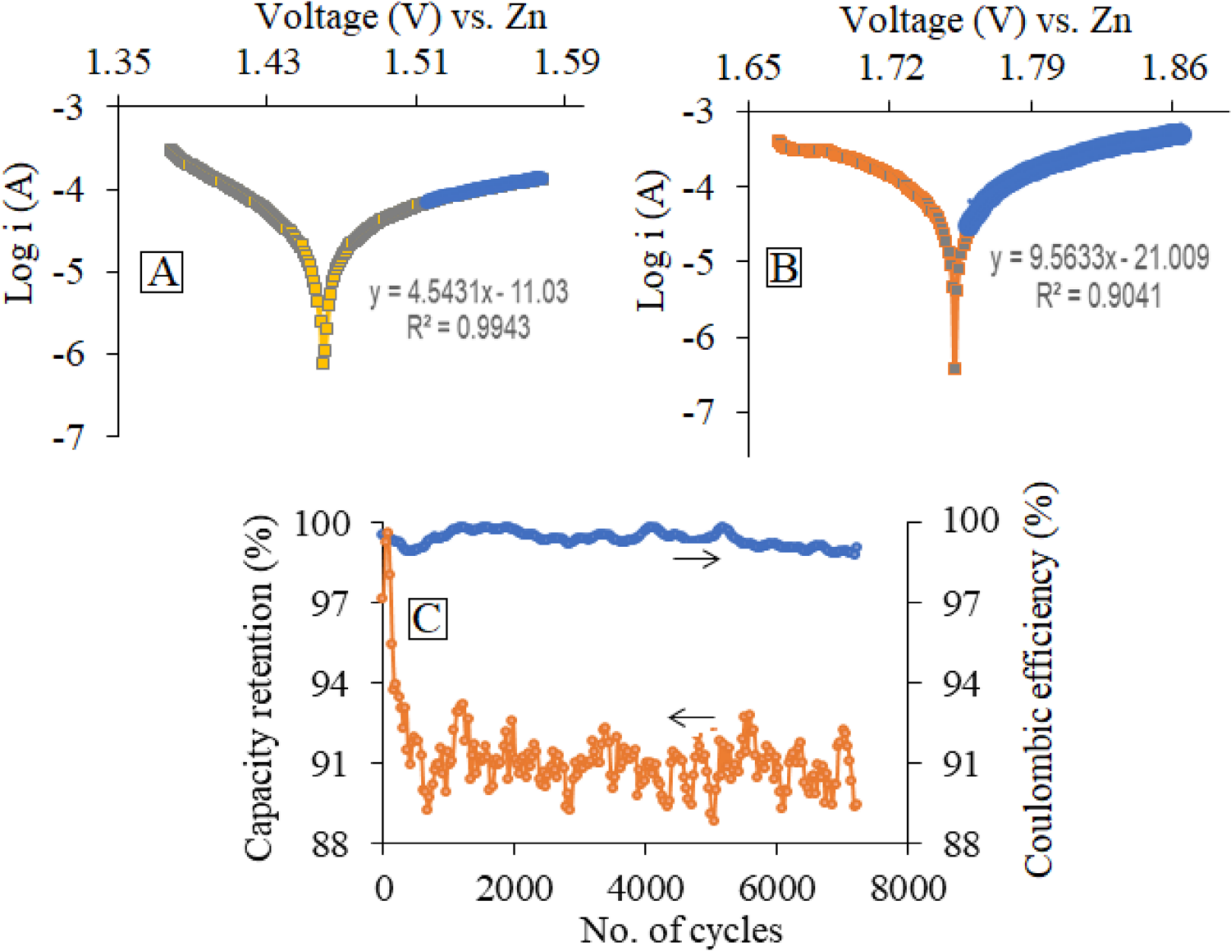
OCP and Tafel plots of the γ-MnO_2_ cathode, zinc anode, and triply-distilled water/ZnSO_4_ (2.0 mol L^−1^) electrolyte in the (**A**) absence, (**B**) presence of tartaric acid (1.0 mol L^−1^, 0.5 mL) as an additive at room temperature and (**C**) capacity retention of the modified ZIB.

However, to further evaluate the ZIB’s behavior before and after introduction with the tartaric acid with 1.0 mol L^−1^ concentration, the correlation between the logarithm of the electrical current and the potential window (between 1.0 and 2.0 V, vs. the Zn) was plotted based on the applied potential at high possible potential range using the *Tafel* equation (Fig. 5).

Correlation between electrical current and the electrical potential, before (Fig. 5A) and after (Fig. 5B) introduction of tartaric acid again indicated that the adopted additive used in this system was very promising for high-performance and capacity retention. As a result, capacity retention (Fig. 5C) of the battery after 7200 cycles at a rate of 5C was estimated to be 91.0%.

Based on the slope, intercept of the linear correlation, net current (*i*_*0*_) as well as transfer coefficient (*α*) of the electrode system in the presence of the electrolyte additive, were found as 5.62 × 10^–5^ A and 0.56, respectively, Whereas, these values were found as 3.57 × 10^–5^ A and 0.27, respectively, in the absence of the electrolyte additive, under similar conditions. Compared to the values estimated in the absence of any additives, these results again exhibited the influential role of tartaric acid for lowering the over-potential of the electrochemical reaction during the promotion of the capability, capacity as well as net electrical potential of the modified ZIB. Enhancement effect of the i_0_ (5.62 × 10^–5^ A) and α (0.56) also revealed the promotion of the efficiency of the ZIB during the additive introduction.

It seems that tartaric acid, by increasing in ionic conductivity of battery electrolyte, leads to the enhancement of electrical current in contrast to the absence of tartaric acid. On the other hand, it can be concluded that tartaric acid can play a vital role in decreasing transfer coefficient (*α*) for charge transfer and intercalation of zinc ions into γ-MnO_2_ layer structure. It seems that tartaric acid can be adsorbed onto the surface of the electrode and prepare a suitable energy active site for intercalation and de-intercalation for ions. Also, it should be noted that MnO_2_ is a ceramic Nanoparticle that cannot have a chemical reaction in an acidic medium.

However, it should be noted that, due to the obstacle of infrared or Raman spectroscopy in the observation of physical interaction, no significant changes were observed in the infrared and/or Raman spectra ea of tartaric acid. On the other hand, MnO_2_ is well known as an inorganic ceramic. In addition, according to much literatures, MnO_2_ cannot has any chemical reaction(s). So, except changing in the electrode surface voltage as illustrated in Fig. 5A,B as well as the BET test, it was impossible for us to express some other proofs. On the other hand, γ-MnO_2_ was synthesized based on the literature, and its XRD spectrum was completely matched by the previous reports.

### Proposed mechanism

**Figure 6:**
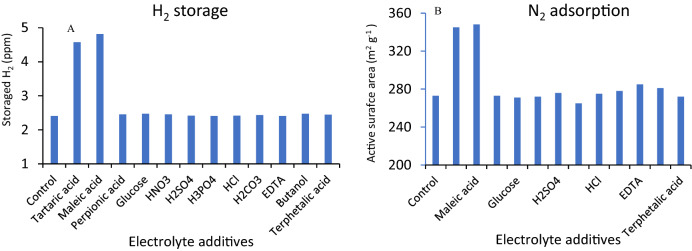
BET analyses different electrolyte additives based on the (**A**) H_2_ storage and (**B**) N_2_ adsorption process during the charging step.

Since γ-MnO_2_ is a ceramic and the ceramics are chemically and electrochemically less reactive^34^, compared to other types of materials, as well as based on the results obtained from *Tafel* plot, electrochemical impedance spectroscopy, and capacity retention tests, some experiments were used to confirm (propose) the following probable mechanism. At first, BET analysis was performed, and various electrolyte additives were used (1.0 mol L^−1^) under similar conditions, as clearly pointed out in Fig. 6A,B during the electrical charging steps. Tartaric acid and maleic acid showed the highest storage and adsorption isotherms on the γ-MnO_2_ cathode surface. Whereas, no significant changes were observed between the storage H_2_ and adsorbed N_2_ during the discharging process under similar conditions.

No significant change(s) was observed on the XPS spectrum and the XRD patterns (Fig. 1A) in the presence and absence of tartaric acid. Especially, the XPS, due to different reasons, such as the very thin surface thickness (depth) of MnO_2_ on the cathode surface, the X-ray photoelectron spectroscopy (XPS) spectrum was very noisy and not-reproducible. Consequently, it was impossible to test the morphology of the synthesized MnO_2_ by other spectroscopic methods such as XPS.

However, at the initial stage, it seemed that the interaction between Zn^2+^ and tartaric acid, was considered the responsible factor, but, according to the similarity of the XRD patterns and the XPS spectra in the presence and absence of tartaric acid as the electrolyte additive as well as at both charges and discharge conditions, pointed to the presence of physical interaction such as surface interaction, which was in good agreement with the BET analyses (Fig. 6). Based on the results, the sequential adsorption/desorption of tartaric acid on the surface cathode, ultimately on the polar functional groups like hydroxyl (−OH) and carboxylic acid *(*−COOH), resulted in the significant enhancement of the double-layer capacitance, while providing a suitable active site for the perching the Zn^2+^ for the intercalation and electrochemical reduction of MnO_2_. These results showed the reversible and smart adsorption/desorption of tartaric acid on the surface of the modified cathode. Therefore, it can be concluded that, these acids were adsorbed on the γ-MnO_2_ edges in reversible Langmuir isotherm mode. Consequently, it is the cause of extended retention capacity, and make a suitable site in terms of energy and surface area.

It should be mentioned that, in addition to the BET test, EIS, linear polarization (Tafel plot) as well as CV were performed, which can be used as an acceptable proof (proposed behavior) for this claim. According to the EIS spectrum, the charge transfer of cathode almost was modified as large as around tenfold. On the other hand, enhancement in double layer capacitance after tartaric acid addition resulted in accessing to a significant value as large as ~ 500-fold. This significant change is considered as another proof for indirectly demonstrating the reversible adsorption of tartaric acid to the cathode interface. In addition to this phenomenon, the transfer coefficient of MnO_2_ as a unique criterion of charge transfer in the interface of electrode/electrolyte was increased approximately twofold (0.56, 0.27 in the presence and absence of tartaric acid, respectively). All these obtained results affirmed the physisorption of tartaric acid onto the electrode surface in this report.

## Conclusions

We fabricated a reversible aqueous Zn/MnO_2_ battery. The modified γ-MnO_2_ cathode was highly reversible and stable in a ZnSO_4_ aqueous electrolyte during using tartaric acid as the electrolyte additive that played the role in reducing the over-voltage. This process, therefore exhibited an excellent capacity as large as 374 mAh g^−1^, rate capability, and high-capacity retention of 91.0% after 7200 cycles. Some comparisons of electrochemical performances between this research and previously reported researches are based on the Zn/MnO_2_ battery (Table 1).

**Table 1:** Comparison of this work with the previously reported ZIBs batteries.

Cathode material	Electrolyte	Average discharge voltage (vs Zn/Zn^2+^)/specific capacity/rate performance	Cycle performance	References
α-MnO_2_	1.0 mol L^−1^ ZnSO_4_	1.26 V at 10.5 mA g^−1^ 205 mAh g^−1^ at 10.5 mA g^−1^ 57.7%—retained at 210 mA g^−1^	66% retained after 30 cycles at 10.5 mA g^−1^	^30^
α-MnO_2_	1.0 mol L^−1^ ZnSO_4_	1.3 V at 10.5 mA g^−1^ 195 mAh g^−1^ at 10.5 mA g^−1^ 85.6% retained at 42 mA g^−1^	70% retained after 30 cycles at 42 mA g^−1^	^35^
α-MnO_2_	2.0 mol L^−1^ ZnSO_4_ + 0.1 mol L^−1^ MnSO_4_	1.32 V at 61.6 mA g^−1^ 255 mAh g^−1^ at 61.6 mA g^−1^ 44.3% retained at 3080 mA g^−1^	92% retained after 5000 cycles at 1540 mA g^−1^	^2^
Todorokite-type MnO_2_	1.0 mol L^−1^ ZnSO_4_	1.3 V at 50 mA g^−1^ 108 mAh g^−1^ at 50 mA g^−1^	83% retained after 50 cycles at 50 mA g^−1^	^36^
γ-MnO_2_	1.0 mol L^−1^ ZnSO_4_	1.32 V at 0.05 mA cm^−2^ 285 mAh g^−1^ at 0.05 mA cm^−2^_,_ 57.8% retained at 0.5 mA cm^−2^	63% retained after 40 cycles at 0.5 mA cm^−2^	^37^
Zn_2_V_2_O_7_	1.0 mol L^−1^ ZnSO_4_	0.68 V at 300 mA g^−1^ 248 mAh g^−1^ at 50 mA g^−1^ 68% retained at 4400 mA g^−1^	85% retained after 1000 cycles at 4000 mA g^−1^	^38^
**γ-MnO**_**2**_	**2.0 mol L**^−**1**^** ZnSO**_**4**_** + tartaric acid (1.0 mol L**^−**1**^**)**	** + 1.46 V (vs. Zn) at 374 mAh g**^−**1**^ **92.1% retaining at 7000 mA g**^−**1**^	**91.0% retaining after 7200 cycles**	**Present study**

Based on this comparison, it was concluded that, we introduced novel types of electrolyte additives to modify the Zn/MnO_2_ battery, which efficiently improved the electrochemical behavior of the battery and brought a longer battery lifetime, significantly. The motivation behind this study results in creating a more efficient, reliable, environmentally friendly, low cost, high enough electrical energy, higher power densities, more improved reversibility, and longer cycle life, compared to the general ZIBs.

## References

[1] Huria, T. & Ceraolo, M. Rechargeable lithium battery energy storage systems for vehicular applications. Ph.D. Thesis. (2012).

[2] Pan H (2016). Reversible aqueous zinc/manganese oxide energy storage from conversion reactions. Nat. Energy.

[3] Miura A (1989). Development of corrosion resistant zinc alloys for alkaline-manganese batteries. A. Miura al Denki Kagaku.

[4] Kalyani P, Chitra S, Mohan T, Gopukumar S (1999). Lithium metal rechargeable cells using Li2MnO3 as the positive electrode. J. Power Sources.

[5] Winter M, Brodd RJ (2004). What are Batteries, Fuel Cells, and Supercapacitors?.

[6] Liu J, Xu C, Chen Z, Ni S, Shen ZX (2018). Progress in aqueous rechargeable batteries. Green Energy Environ..

[7] Kim H, Hong J, Park K-Y, Kim H, Kim S-W, Kang K (2014). Aqueous rechargeable Li and Na ion batteries. Chem. Rev..

[8] Pasta M, Wessells CD, Huggins RA, Cui Y (2012). A high-rate and long cycle life aqueous electrolyte battery for grid-scale energy storage. Nat. Commun..

[9] Lu Y, Goodenough JB, Kim Y (2011). Aqueous cathode for next-generation alkali-ion batteries. J. Am. Chem. Soc..

[10] Liu Z, Pulletikurthi G, Endres F (2016). A prussian blue/zinc secondary battery with a bio-ionic liquid–water mixture as electrolyte. ACS Appl. Mater. Interfaces.

[11] Kundu D, Adams BD, Duffort V, Vajargah SH, Nazar LF (2016). A high-capacity and long-life aqueous rechargeable zinc battery using a metal oxide intercalation cathode. Nat. Energy.

[12] Müller-Warmuth W, Schöllhorn R (2012). Progress in Intercalation Research.

[13] Kang K, Meng YS, Bréger J, Grey CP, Ceder G (2006). Electrodes with high power and high capacity for rechargeable lithium batteries. Science (80-).

[14] Xu C, Li B, Du H, Kang F (2012). Energetic zinc ion chemistry: The rechargeable zinc ion battery. Angew. Chem. Int. Ed..

[15] Ozoemena KI, Chen S (2016). Nanomaterials in Advanced Batteries and Supercapacitors.

[16] Fang G, Zhou J, Pan A, Liang S (2018). Recent advances in aqueous zinc-ion batteries. ACS Energy Lett..

[17] Guo S (2021). Fundamentals and perspectives of electrolyte additives for aqueous zinc-ion batteries. Energy Storage Mater..

[18] Guo X (2021). Alleviation of dendrite formation on zinc anodes via electrolyte additives. ACS Energy Lett..

[19] Seki S (2007). Imidazolium-based room-temperature ionic liquid for lithium secondary batteries effects of lithium salt concentration. J. Electrochem. Soc..

[20] Wright PV (2002). Developments in polymer electrolytes for lithium batteries. Mrs Bull..

[21] Hou Z, Zhang X, Li X, Zhu Y, Liang J, Qian Y (2017). Surfactant widens the electrochemical window of an aqueous electrolyte for better rechargeable aqueous sodium/zinc battery. J. Mater. Chem. A.

[22] Xu W (2019). Diethyl ether as self-healing electrolyte additive enabled long-life rechargeable aqueous zinc ion batteries. Nano Energy.

[23] Lee J (2011). Metal–air batteries with high energy density: Li–air versus Zn–air. Adv. Energy Mater..

[24] Wu B (2018). Graphene scroll-coated α-MnO_2_ nanowires as high-performance cathode materials for aqueous Zn-ion battery. Small.

[25] Fu Y (2018). High-performance reversible aqueous Zn-ion battery based on porous MnOx nanorods coated by MOF-derived N-doped carbon. Adv. Energy Mater..

[26] Huang J (2018). Polyaniline-intercalated manganese dioxide nanolayers as a high-performance cathode material for an aqueous zinc-ion battery. Nat. Commun..

[27] Liu G, Huang H, Bi R, Xiao X, Ma T, Zhang L (2019). K^+^ pre-intercalated manganese dioxide with enhanced Zn 2+ diffusion for high rate and durable aqueous zinc-ion batteries. J. Mater. Chem. A.

[28] Liu Z (2021). Improving stability and reversibility via fluorine doping in aqueous zinc–manganese batteries. Mater. Today Energy.

[29] Zhang SS (2006). A review on electrolyte additives for lithium-ion batteries. J. Power Sources.

[30] Alfaruqi MH (2015). Electrochemically induced structural transformation in a γ-MnO_2_ cathode of a high capacity zinc-ion battery system. Chem. Mater..

[31] Zhang N (2017). Rechargeable aqueous zinc-manganese dioxide batteries with high energy and power densities. Nat. Commun..

[32] Vondrák, J. “*Electrochemical methods: Fundamentals and applications: by AJ Bard and LR Faulkner; published by Wiley, New York, 1980; xviii+ 718 pp.; price, £ 33.65 (hard cover), £ 13.00 (soft cover); ISBN 0-471-05542-5*”. (Elsevier, 1983).

[33] Darkrim FL, Malbrunot P, Tartaglia GP (2002). Review of hydrogen storage by adsorption in carbon nanotubes. Int. J. Hydrogen Energy.

[34] Ricoult MB, Badding M, Thibault Y (2005). Advances in electronic and electrochemical ceramics. Ceram. Trans.

[35] Lee B, Lee HR, Kim H, Chung KY, Cho BW, Oh SH (2015). Elucidating the intercalation mechanism of zinc ions into α-MnO_2_ for rechargeable zinc batteries. Chem. Commun..

[36] Lee J, Ju JB, Il Cho W, Cho BW, Oh SH (2013). Todorokite-type MnO_2_ as a zinc-ion intercalating material. Electrochim. Acta.

[37] Guo X (2018). A hollow-structured manganese oxide cathode for stable Zn-MnO_2_ batteries. Nanomaterials.

[38] Sambandam B (2018). Aqueous rechargeable Zn-ion batteries: An imperishable and high-energy Zn_2_V_2_O_7_ nanowire cathode through intercalation regulation. J. Mater. Chem. A.

